# A single-arm, open-label, intervention study to investigate the improvement of glucose tolerance after administration of the 5-aminolevulinic acid (5-ALA) in the patients with mitochondrial diabetes mellitus

**DOI:** 10.1097/MD.0000000000025100

**Published:** 2021-03-12

**Authors:** Yuta Nakamura, Ai Haraguchi, Riyoko Shigeno, Ayako Ito, Ichiro Horie, Atsushi Kawakami, Norio Abiru

**Affiliations:** a Department of Endocrinology and Metabolism, Nagasaki University Hospital; b Department of Endocrinology and Metabolism, Division of Advanced Preventive Medical Sciences, Nagasaki University Graduate School of Biomedical Sciences, Nagasaki, Japan.

**Keywords:** 5-aminolevulinic acid/sodium ferrous citrate, insulin secretory capacity, mitochondrial diabetes mellitus, open-label

## Abstract

**Background:**

Mitochondrial diabetes mellitus (MDM) is characterized by maternal inheritance, progressive neurosensory deafness, insulin secretory disorder, and progressive microvascular complications. Mitochondria are critical organelles that provide energy in the form of adenosine triphosphate (ATP). An impairment of ATP production in pancreatic β cells is regarded as the main cause of the insulin secretory disorder in patients with MDM, and these patients require insulin replacement therapy early after the diagnosis. The amino acid 5-aminolevulinic acid (5-ALA), a precursor of heme metabolites, is a non-proteinogenic δ amino acid synthesized in mitochondria. An addition of ferrous iron to 5-ALA enhances heme biosynthesis and increases ATP production through an upregulation of the respiratory complex. Several studies have reported that the administration of 5-ALA and ferrous iron to existing treatment improved the glycemic control in both patients with prediabetes and those with type 2 diabetes mellitus. The additional administration of 5-ALA and ferrous iron to MDM patients on insulin therapy may improve their insulin secretory capacity and glycemic control by improving their mitochondrial function. The findings of this study are expected to provide new treatment options for MDM and improve the patients’ glycemic control and prognosis.

**Methods/design:**

This study is a single-arm, open-label pilot intervention study using clinical endpoints to investigate the effects of treatment with 5-ALA plus sodium ferrous citrate (SFC) to patients with MDM on their glucose tolerance. A total of 5 patients with MDM will be administered 5-ALA/SFC (200 mg/d) for 24 weeks. We will perform a 75-g oral glucose tolerance test before and at 24 weeks after the start of this 5-ALA/SFC treatment to evaluate glucose-dependent insulin responses.

**Discussion:**

To the best of our knowledge, this study will be the first assessment of the effects of 5-ALA/SFC in patients with MDM. This study will obtain an evidence regarding the effectiveness and safety of 5-ALA/SFC for patients with MDM.

**Trial registration:**

This study was registered with the University Hospital Medical Information Network (UMIN000040581) on July 1, 2020 and with the Japan Registry of Clinical Trials (jRCTs071200025) on August 3, 2020.

## 1 Introduction

Mitochondrial diseases are heterogeneous disorders characterized by defects of energy production via oxidative phosphorylation. Endocrine dysfunction is often observed in genetic mitochondrial diseases, and among those diseases, diabetes mellitus is the most frequently described as mitochondrial diabetes mellitus (MDM). The m.3243 A>G mutation is the most common point mutation of the mitochondrial deoxyribonucleic acid (mtDNA) observed in patients with MDM. However, it might be impossible to identify the mutation of the mtDNA using the peripheral leukocytes in all patients with MDM, because heteroplasmy values show large distributions between individual tissues. It was reported that only 13.1% of the blood samples were positive for the m.3243 A>G mutation among Japanese patients with MDM.^[1]^ Generally, MDM has the following characteristics.

(1) It is maternally inherited;
(2) it requires insulin therapy due to the progressive insulin secretory disorder;
(3) it shows progressive neurosensory deafness; and
(4) it shows rapidly progressive diabetic complications.^[1–4]^

The current recommended treatment for MDM is only insulin replacement therapy.

Mitochondria are critical organelles that provide energy in the form of adenosine triphosphate (ATP) for the insulin secretory reaction by glucose stimulation. Mitochondrial dysfunction triggers the development of diabetes mellitus by an impairment of ATP production, which is highly dependent on adequate heme synthesis.^[5]^

The amino acid 5-aminolevulinic acid (5-ALA), a nonproteinogenic δ amino acid found in the human body, promotes intracellular energy metabolism, and the addition of ferrous iron to 5-ALA enhances the biosynthesis of heme, which acts as a protein-bound prosthetic group in mitochondrial respiratory chain complexes II, III, and IV and cytochrome c. The administration of 5-ALA combined with sodium ferrous citrate (SFC) enhance heme production, leading to respiratory complex upregulation.^[6]^ Thus, 5-ALA/SFC might be effective for improving mitochondrial dysfunction and increasing the production of ATP. Treatment with 5-ALA/SFC might also improve insulin secretion by improving mitochondrial dysfunction. In fact, several studies showed beneficial effects of 5-ALA on glycemic control in both prediabetic and diabetic patients.^[7–10]^

We hypothesized that adding 5-ALA/SFC to insulin treatment in patients with MDM may improve insulin secretion and glycemic control. We, therefore, designed this study as the first step in testing the effectiveness of 5-ALA/SFC as a treatment for MDM. Herein, we describe the final study protocol (version 1.0; April 24, 2020). The results of this study are expected to provide evidence of the usefulness of 5-ALA/SFC in the treatment of patients with MDM.

## 2 Methods/design

### 2.1 Objective

This is a single-arm, open-label, pilot intervention study to investigate the effects of 5-ALA/SFC in patients with MDM. We created the present study design in accordance with Standard Protocol Items: Recommendations for Interventional Trials and Consolidated Standards of Reporting Trials 2010 guidelines.^[11,12]^

### 2.2 Study design

The study will be conducted at the Nagasaki University Hospital in Japan. In total, 5 patients with MDM will be recruited to the study, with a 24-week duration of intervention.

### 2.3 Ethical considerations

The study has been approved by the Certified Review Board (CRB) of Nagasaki University (CRB approval no.: CRB20-012) and is registered in the Japan Registry of Clinical Trials (https://jrct.niph.go.jp) as jRCTs071200025. The study will be conducted in accordance with the principles of the Declaration of Helsinki,^[13]^ Japan's Clinical Trials Act (Act No. 16 of April 14, 2017), Japan's Act on the Protection of Personal Information and related regulatory notifications, and the present study protocol.

### 2.4 Study endpoints

#### 2.4.1 Primary endpoint

Changes in glucose-stimulated insulin secretion after the daily administration of 5-ALA/SFC for 24 weeks. To determine the changes, the values of area under the curve of serum insulin levels from fasting (0 minutes) to 120 minutes during 75-g oral glucose tolerance test (OGTT) evaluated at 24 weeks after the administration of 5-ALA/SFC (week 24) will be compared to those at baseline (week 0).

#### 2.4.2 Secondary endpoints

Changes in the following items after the daily administration of 5-ALA/SFC for 24 weeks: fasting blood glucose, glycated hemoglobin, glycated albumin, values obtained from the 75-g OGTT, values obtained from the flash glucose monitoring (FGM), and the required daily dose of insulin. For each parameter, any change will be determined based on the difference between the results at baseline (week 0) and those at week 24.

#### 2.4.3 Safety endpoints

All adverse events that occurred during the trial will be recorded.

### 2.5 Participant recruitment

Participants will be recruited at the Nagasaki University Hospital. The study will be fully explained to potential participants by investigators; participants will then be asked to voluntarily sign an informed consent form before their participation.

### 2.6 Inclusion criteria

The study inclusion criteria are as follows:

(1) Patients aged ≥20 years at the time of consent
(2) Outpatients
(3) Patients diagnosed with MDM who satisfy all of the following main criteria and one or more of the sub-criteria

Main criteria

(1) Patients with diabetes mellitus being treated with insulin or clinically requiring insulin therapy for glycemic control due to decreased insulin secretion
(2) Patients complicated with neurosensory deafness

Sub-criteria

(1) Patients who have a family history of maternally inherited diabetes mellitus
(2) Patients who show a level of serum lactate ≥ 18 mg/dL (2 mmol/L) and/or a molar ratio of lactate to pyruvate ≥20
(3) Patients with short stature whose height is less than –2.0 standard deviation of Japanese adults
(4) Patients complicated with cardiomyopathy or cardiac conduction abnormality
(5) Patients whose basal-ganglia and brainstem have bilaterally symmetric abnormal lesions on computed tomography/magnetic resonance imaging
(6) Patients who have had not changed their antidiabetic agent(s) expect for insulin within 8 weeks before the informed consent is obtained
(7) Written informed consent

### 2.7 Exclusion criteria

The study exclusion criteria are as follows:

(1) Patients who have previously taken 5-ALA/SFC within 8 weeks before obtaining an informed consent
(2) Patients treated with metformin
(3) Patients with a history of porphyria, hemochromatosis, or viral hepatitis
(4) Women who are currently pregnant or lactating
(5) Patients will not be compliant with a medically approved contraceptive regimen during the study period
(6) Patients who participated in another clinical study within the past 4 months
(7) Patients whom the investigators consider inappropriate for participation

### 2.8 Withdrawal criteria

- A patient may be withdrawn from the study prematurely for the following reasons:
- The patient asks to leave the trial.
- Continuing participation is inadvisable due to one or more adverse events.
- 5-ALA/SFC must be withdrawn for ≥4 weeks for any reason(s).
- The patient becomes pregnant.
- In the investigator's opinion, continuation in the trial would be detrimental to the patient's well-being.

### 2.9 Study protocol

**Figure 1:**
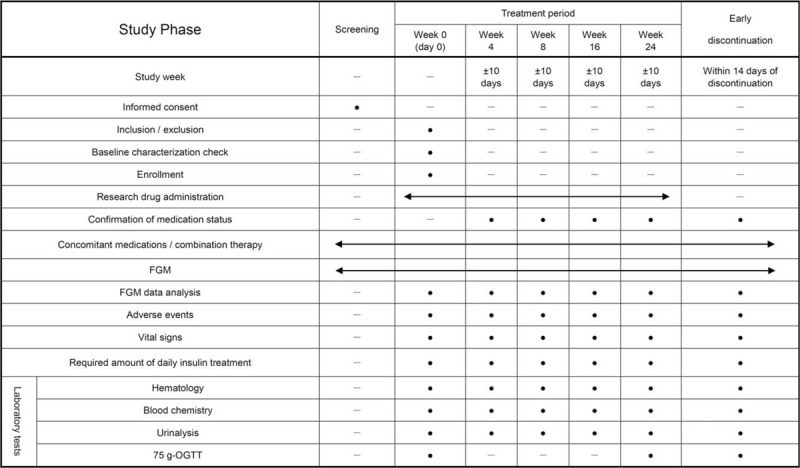
Treatment schedule and outcome measures. FGM = flash glucose monitoring, OGTT = oral glucose tolerance test.

Participants with MDM will be administered 5-ALA/SFC 100 mg twice a day (200 mg/d) for 24 weeks. An investigator will explain the study protocol to each patient and, if consent is obtained, the investigator will perform the observation/examination at the time of registration in accordance with the description in Figure 1. According to the inclusion and exclusion criteria, the investigator will submit the participant's registration form to the study secretariat at the Department of Endocrinology and Metabolism, Nagasaki University Hospital.

After the visit date, the investigator will continue to administer 5-ALA/SFC and conduct necessary examinations and surveys until week 24 in accordance with the schedule in Figure 1. The investigator will refer to the patient's FGM result to adjust the insulin dose based on his/her own judgement. During the study period, any change of the drug or dose of antidiabetic agents (except insulin) is prohibited.

To evaluate the patients’ glucose-dependent insulin responses, a 75-g OGTT will be performed twice: before the start of the 5-ALA/SFC regimen (week 0, 1st OGTT) and at 24 weeks after the start of the 5-ALA/SFC regimen (week 24, 2nd OGTT). When patients need to be withdrawn from the study for any reason, they will undergo 75-g OGTT after discontinuing the 5-ALA/SFC. Both OGTTs will be carried out under the condition of overnight fasting. All antidiabetic agents including insulin and 5-ALA/SFC will be held until the preceding day in each OGTT.

### 2.10 Laboratory measurements

The 2 OGTTs will use a 75-g glucose formulation, Trelan-G75 (AY Pharma, Tokyo). For each OGTT, the levels of plasma glucose (mg/dL), serum insulin (μU/mL), serum C-peptide (ng/mL), and plasma glucagon (pg/mL) will be measured at fasting (0 minutes) and at 30, 60, 120 minutes after the ingestion of the glucose load. We will also measure the serum levels of glycated albumin, nonesterified fatty acid, leptin, adiponectin, high-molecular-weight adiponectin, iron, unsaturated iron-binding capacity, and ferritin at 0 minutes. Urine specimens will be obtained on each OGTT day for the measurement of the patient's albumin/creatinine ratio.

The levels of serum insulin and C-peptide will be measured by the ECLusys kit (Roche, Basel, Switzerland). Blood samples for plasma glucagon will be obtained using BD P800 tubes (BD, Franklin Lakes, NJ). Plasma glucagon will be measured by a sandwich ELISA kit (Mercodia, Uppsala, Sweden).

As shown in Figure 1, we will measure the patient's complete blood count and serum (plasma) levels of sodium, potassium, chlorine, total cholesterol, high-density lipoprotein, cholesterol, low-density lipoprotein, blood urea nitrogen, creatinine, total bilirubin, aspartate aminotransferase, alanine aminotransferase, alkaline phosphatase, γ-glutamyl transpeptidase, creatine kinase, glucose, and glycated hemoglobin at every visit of the study (week 0, 4, 8, 16, and 24), and (if withdrawing from the study) at early discontinuation.

### 2.11 Adverse events

A serious adverse event is defined as any untoward medical event that occurs at any dose, results in death, a life-threatening condition, requires hospitalization, and results in significant disability or incapacity.

All serious adverse events occurring between the signing of the informed consent form and the end of the trial will be documented in the medical records and reported to the Minister of Health, Labor, and Welfare (Japan) and the CRB by the responsible investigator in accordance with Japanese regulations.

### 2.12 Data collection and management

The sponsor-investigator and sub-investigators will fill in all of each patient's data in a case report form (CRF). It is necessary that all data recorded in the CRF are consistent with the original material, unless data recorded directly in the CRF are used as the source material. For the results of FGM, the output data signed by the investigator will be regarded as the original material. During the study, the investigators will collect data at each visit in accord with the schedule in Figure 1. All data recorded in the CRF will be checked by the data manager and will then be fixed/set. All study findings and documents will be made confidential, and patients will always be identified by their patient number, never by name. Confidential patient-identifying documents will be maintained by the investigators to preserve participant anonymity.

During the study, a sponsor-investigator will perform regular site visits to review the protocol compliance, conduct source data verification, assess drug accountability and management, and assess laboratory procedures to ensure that the study is being conducted according to all pertinent requirements.

### 2.13 Statistical analysis

The respective analysis sets are defined as follows. The intention to treat (ITT) population is defined as all participants registered for this trial; the safety analysis set (SAS) population is defined as the participants in the ITT population with at least 1 administration of 5-ALA/SFC. The full analysis set (FAS) population is defined as the participants in the SAS for whom data after the administration of 5-ALA/SFC at one or more scheduled visits are available. The per protocol set (PPS) is defined as the participants in the FAS with the primary outcome measure.

The characteristics of all 5-ALA/SFC-treated populations will be summarized using descriptive statistics on the ITT, SAS, FAS, and PPS.

An efficacy analysis will be conducted using the FAS and PPS. The primary endpoint and secondary endpoints listed above in Section 2.4 will be estimated as point estimates and 95% confidence intervals and summarized using descriptive statistics.

Safety endpoints will be analyzed in the SAS population. Adverse events will be stratified by serious versus nonserious, causal relationship, severity, and outcome. Analyses will be categorized and tabulated, and a list will be formulated including causality, extent, and outcomes.

Data containing missing values will be also included in the analysis. Missing values will be imputed by a last observation carried forward analysis. All data will be analyzed after being fixed/set. All tests of significance will be done at a 2-tailed significance level of 0.05. Data relevant to this clinical trial will be analyzed using SAS, SPSS, R software, or JMP Pro 15 or higher.

## 3 Discussion

A nationwide case-finding study of genetically defined MDM patients in Japan revealed that 86% of the patients required insulin therapy due to the progressive insulin secretory disorder, and 92% of them had sensorineural deafness.^[1]^ Individuals with MDM have advanced microvascular complications and mitochondria-related complications such as cardiomyopathy, cardiac conductance disorders, neuromuscular symptoms, neuropsychiatric disturbance, and macular pattern dystrophy caused by glycemic variability and higher oxidative stress levels.^[14,15]^ To prevent the development of those complications, therapy in addition to insulin has been desired to directly improve the mitochondrial dysfunction in patients with MDM.

Diagnostic criteria for mitochondrial disease were defined by Japan Intractable Diseases Information Center,^[16]^ but criteria that are specialized for MDM have not been established. We set main criteria as described in the inclusion criteria above based on the results of the nationwide case-finding study.^[1]^ We also set the sub-criteria described above since that study also reported that the rate of a family history of maternal inheritance was 68%,^[1]^ and the Japanese criteria for mitochondrial disease include items such as a high serum lactate level, short stature, cardiomyopathy or cardiac conduction abnormality, and bilateral symmetric abnormal lesions in the basal ganglia and/or brainstem on computed tomography/magnetic resonance imaging.

The administration of 5-ALA to healthy mice activate their mitochondrial respiratory function by increasing the protein expression and activity of cytochrome c oxidase (complex IV).^[6]^ In addition, Shimura et al. recently reported that 5-ALA/SFC increased the oxygen consumption rate and ATP level via an oxidative phosphorylation protein's expression induced by upregulations of heme, heme oxygenase-1, and the mtDNA copy number in skin fibroblasts from individuals with mitochondrial disease.^[17]^

The administration of 5-ALA/SFC reduced blood glucose levels and improved glucose tolerance in diabetic rodents by enhancing mitochondrial function.^[18,19]^ In prediabetic and diabetic human subjects, the oral administration of 5-ALA/SFC also improved their glucose tolerance, although the dosage regimen of 5-ALA/SFC in those studies was lower than that of our present investigation.^[7–10]^ Our study could thus be expected to observe improved effects on insulin secretory capacity and blood glucose regulation by a recovery of the mitochondrial function. For the evaluation of these effect, we will perform a 75-g OGTT before and at 24 weeks after the start of the administration of 5-ALA/SFC and then compare the difference in insulin secretion.

The dosage of the investigational drug will be set at 100 mg taken orally twice daily (200 mg/d) in accord with an ongoing clinical trial for mitochondrial disease. That clinical trial was registered in the Center for Clinical Trials, Japan Medical Association (http://www.jmacct.med.or.jp) as JMA-IIA00358. Another study conducted in patients with type 2 diabetes (ClinicalTrials.gov NCT02481141) demonstrated the safety and tolerability of the dose.^[10]^ In studies using a similar dose of 5-ALA, the adverse events were consistently mild, and some studies did not observe any side effects. We consider that the 200 mg/d dose is acceptable and effective for patients with MDM.

There are some limitations in this trial.

(1) It will be conducted at a single center and the sample size is small (n = 5).
(2) It is a single-arm study and lacks a placebo control group.
(3) The follow-up period of the trial may be insufficient to lead to a definitive conclusion regarding the improvement of insulin secretion and glycemic control.

Despite these limitations, our research also has strengths. There has been no study assessing the effect of 5-ALA/SFC on insulin secretion and glycemic control in patients with MDM. We may discover an effective antidiabetic agent for MDM according to the pathology.

This trial will evaluate the effectiveness and safety of 5-ALA/SFC in patients with MDM. It will test the potential effectiveness of 5-ALA/SFC as adjunctive therapy for patients with MDM receiving insulin therapy. The findings of the study are expected to provide new treatment options for MDM and improve the glycemic control and prognosis of patients with this disorder.

## Acknowledgments

The authors thank their colleagues and staff at the Department of Nagasaki University Hospital for their support.

## Author contributions

**Conceptualization:** Ai Haraguchi, Atsushi Kawakami, Norio Abiru.

**Data curation:** Ai Haraguchi.

**Formal analysis:** Yuta Nakamura, Ichiro Horie, Norio Abiru.

**Funding acquisition:** Norio Abiru.

**Investigation:** Yuta Nakamura, Ai Haraguchi, Riyoko Shigeno, Ayako Ito, Ichiro Horie, Norio Abiru.

**Project administration:** Norio Abiru.

**Writing – original draft:** Yuta Nakamura, Ai Haraguchi.

**Writing – review & editing:** Ichiro Horie, Norio Abiru.
